# Mixotrophic flagellate ingestion boosts microplastic accumulation in ascidians

**DOI:** 10.1002/jez.2596

**Published:** 2022-04-13

**Authors:** Roberta Pennati, Chiara Castelletti, Marco Parolini, Giorgio Scarì, Silvia Mercurio

**Affiliations:** ^1^ Department of Environmental Science and Policy Università degli Studi di Milano Milan Italy; ^2^ Department of Biosciences Università degli Studi di Milano Milan Italy

**Keywords:** algae, bioaccumulation, *Ciona intestinalis*, cryptomonad, trophic transfer, tunicate

## Abstract

Microplastics are contaminants of global environmental concern. They can be ingested by a variety of organisms when they enter the food web. Several studies have reported trophic transfer of microplastics from low trophic levels to higher ones. Bioaccumulation has been suggested to occur but few studies have demonstrated it for marine environments. In this article, in controlled laboratory conditions, we exposed filter‐feeder ascidian juveniles to microplastics in the presence or in absence of mixotrophic cryptomonad flagellates. Cryptomonads can efficiently ingest microbeads, and their presence significantly increased the concentration of microplastics in the digestive tract of the ascidians. Our results demonstrate the occurrence of microplastic bioaccumulation in the lower levels of the marine trophic chain and suggest that unicellular organisms can be key actors in microplastic trophic transfer at the microscale level.

## Research Highlights

• Marine mixotrophic cryptomonad flagellates can ingest microbeads of polystyrene with a diameter of 10 µm.

• The copresence of cryptomonads and microbeads significantly increased the microplastics in the digestive tract of filter‐feeding ascidians.

## Graphical abstract

Mixotrophic flagellate presence boosts microplastic accumulation in ascidian juveniles.

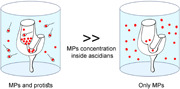

## 1 INTRODUCTION

Microplastics (MPs, i.e., plastic items < 5 mm in size) pollution has become a global environmental issue (Yu et al., 2020). Due to their small size and variable buoyancy, MPs can be ingested by a wide range of organisms across different trophic levels, and they can be transferred along the food web. It has been shown that organisms at lower trophic levels, such as micro‐ and macrozooplankton, can ingest microplastics directly, since the fragments match the size of their preys, and then the animals themselves can be ingested by their predators (Farrell & Nelson, 2013; Gouin, 2020). Indeed, the trophic transfer has been shown to occur in marine ecosystems (Setälä et al., 2014), while bioaccumulation has been only suggested. Bioaccumulation occurs when the uptake of a contaminant is greater than the ability of an organism to egest the contaminant alone (Wang et al., 2016). Biomagnification takes place when an organism faces an increase in the concentration of a contaminant compared with its prey (Miller et al., 2020). As MP bioaccumulation is likely to occur across the marine food web, in this study we exploited our well‐established ascidian model (Messinetti et al., 2018a, 2019) to investigate its occurrence. In particular, we tested whether the presence of cryptomonad mixotrophic flagellates could influence the concentration of MPs ingested by juveniles of the ascidian *Ciona intestinalis*.

Ascidians are sessile filter‐feeding tunicates, a group that is evolutionarily close to vertebrates (Delsuc et al., 2006). In several marine environments, they are essential members of benthic communities, since they can colonize various natural and artificial substrates, thus increasing ecosystem biodiversity (Sepúlveda et al., 2014). They have been extensively exploited as experimental models for ecotoxicological analysis thanks to their amenability in laboratory conditions (Eliso et al., 2020; Messinetti et al., 2018b). Recently, we demonstrated that juveniles of the ascidian *C. intestinalis* could efficiently ingest polystyrene microspheres of 10 µm diameter (Messinetti et al., 2018a); moreover, smaller microbeads (1 µm) could translocate from the intestine lumen into circulating fluid (Messinetti et al., 2019).

Cryptomonads are a relatively small, taxonomically uncertain group of freshwater and marine phytoplankton, which has ecological and evolutionary importance. The members of this group are characterized by the presence of two slightly unequal flagella emerging from the vestibular region where there is a groove, called “gullet,” lined with trichocysts (Santore, 1985; Tranvik et al., 1989). Some cryptomonads are not photosynthetic but are heterotrophic, whereas many photosynthetic species retain the ability to ingest prey and are thus defined as mixotrophic (Novarino, 2005). It has been proposed that photosynthetic microorganisms have to ingest organic particles because they need to obtain nitrogen, phosphorus, and organic trace nutrients such as vitamins (Sanders & Porter, 1988). Generally, in the environment cryptomonads ingest bacteria, but in laboratory conditions, the ingestion of MPs has been reported in the mixotrophic species *Cryptomonas ovata* and *C. erosa* exposed to 0.57 µm diameter polystyrene beads (Tranvik et al., 1989). This ability coupled with their amenability to being cultured and manipulated under laboratory conditions made them an optimal model for our experimental plan. Furthermore, cryptomonads are widespread in the marine environment and abundant along the coastline (Astudillo et al., 2014; Xing et al., 2008) where ascidians, such as *C. intestinalis*, are present (Bouchemousse et al., 2016; Locke & Carman, 2009).

This study would represent the first attempt to experimentally demonstrate MP accumulation in the lower levels of the marine trophic web involving photosynthetic microorganisms.

## 2 MATERIAL AND METHODS

### 2.1 Animals

Adults of *C. intestinalis* were collected by the fishing service of the Station Biologique de Roscoff (France). The animals were kept in aquaria filled with artificial seawater (ASW; Instant Ocean 32‰) at 18 ± 1°C and in constant light condition. To obtain juveniles, gametes were obtained by the dissection of three adults. Cross‐fertilization was performed and embryos were reared until they reached the larval stage at 18°C. Swimming larvae were transferred in glass Petri dishes (Ø = 4 cm), 40 larvae per dish, and allowed to adhere and undergo metamorphosis. After 2 days, metamorphosed juveniles were checked upon; unhealthy small ones were discarded while the others were reared in filtered artificial seawater buffered with 5 mM Hepes pH 8 (ASWH) until they reached the developmental stage 8 (2nd ascidian stage) (Chiba et al., 2004). Ascidian juveniles were fed daily with a commercial algal suspension (Phyto Reef, SHG) and the seawater was replaced every 2 days.

Cryptomonad flagellates from Mediterranean Sea were cultured in glass flasks in natural light until they reached the concentration of about 10,000 cells/ml. Cell density was determined using Benton and Dickinson (Aria I) Fluorescent Activated Cell Sorter (BD FACS Aria II).

### 2.2 Microplastics

Red polystyrene microbeads with a nominal particle diameter of 10 μm were purchased from Sigma‐Aldrich. The commercial standard was an aqueous suspension with a particle concentration of 50 mg/ml. 75 µl of this stock suspension were diluted in 2.5 ml of ASWH to obtain a 1.5 µg/µl suspension, which was used for the experiments. The concentration of MPs in this solution was estimated to be approximately 2000 MPs/ml (1981 ± 79 MPs/ml) since the particle specific gravity indicated by the manufacturer was 1.51 g/cm3 and the calibrated particle diameter was 9.86 ± 0.13 µm.

### 2.3 Exposure

All of the experimental procedures were performed at 18 ± 1°C. Five ml of cryptomonads culture were mixed with 5 ml of diluted red MPs, reaching the final concentration of 0.725 µg/µl (~1000 MP/ml). The flagellates were kept for 30 min in this medium, observed and photographed under a Leica transmission microscope. Then, 0.5 ml of the mixture was fixed adding 0.5 ml of 8% paraformaldehyde in phosphate‐buffered saline (PBS) to determine the percentage of cells that phagocyted MPs according to Heinzelmann et al. (1999). The suspension of cryptomonads was centrifuged and washed with PBS buffer, then the solution was centrifuged, and the sediment was washed by alcohol 20% to 70% for several times to remove salts. This suspension was used for flow cytometry analysis (Croce & Bottiroli, 2014; Surre et al., 2018). A FACScan emitting an argon laser beam at 558 nm (Becton Dickinson BD FACS Aria II Systems) was used to detect forward scatter (FSC), fluorescence 1 (FL1‐H, green), and fluorescence 2 (FL2‐H, red), respectively. Side scatter signal Vs FL2 parameter (emitted fluorescence intensity of red‐stained MPs) were used in the analysis.

Meanwhile, ascidian juveniles were gently detached from the Petri dishes with a needle and transferred in 49 small glass beakers (Ø = 2 cm) containing 800 µl of ASWH. A juvenile was put in each beaker and allowed to recover for 40 min. Then, 200 µl of the previous mixture of flagellates and MPs were added to 26 beakers, to reach the final concentrations of 0.15 µg/µl of MPs (~200 MP/ml), and approximately 500 cells/ml of flagellates. As showed by flow cytometry analysis, part of MPs were ingested by the flagellates and part were free in the medium (see Section 3) and we assumed that the overall concentration of MPs (inside the flagellates fraction plus free in the medium fraction) was that obtained by the serial dilutions.

In the remaining 23 beakers, 100 µl of diluted MPs and 100 µl of ASWH were added to obtain the same final MP concentration of 0.15 µg/µl.

Based on preliminary trials and published works (Messinetti et al., 2019), exposure lasted 1.5 h and after this period of time juveniles were fixed in 4% paraformaldehyde in PBS for 1 h. After a few rinses in PBS, the samples were mounted on glass slides and observed under a transmission microscope. The number of MPs in the digestive tract was annotated for each sample.

### 2.4 Statistical analysis

A generalized linear mixed‐effects model including the treatment, in terms of administration of MPs in a medium with or without flagellates, as fixed factor and the identity of the exposure tank as a random factor was run to investigate changes in ingestion of MPs by *C. intestinalis* specimens. A Poisson distribution of the data was assumed. The significance of results was tested by a likelihood ratio test (LRT). Statistical analyses were performed in the R 3.6.1 environment using nlme package (R Core Team, 2019).

## 3 RESULTS

### 3.1 Phagotrophy

**Figure 1:**
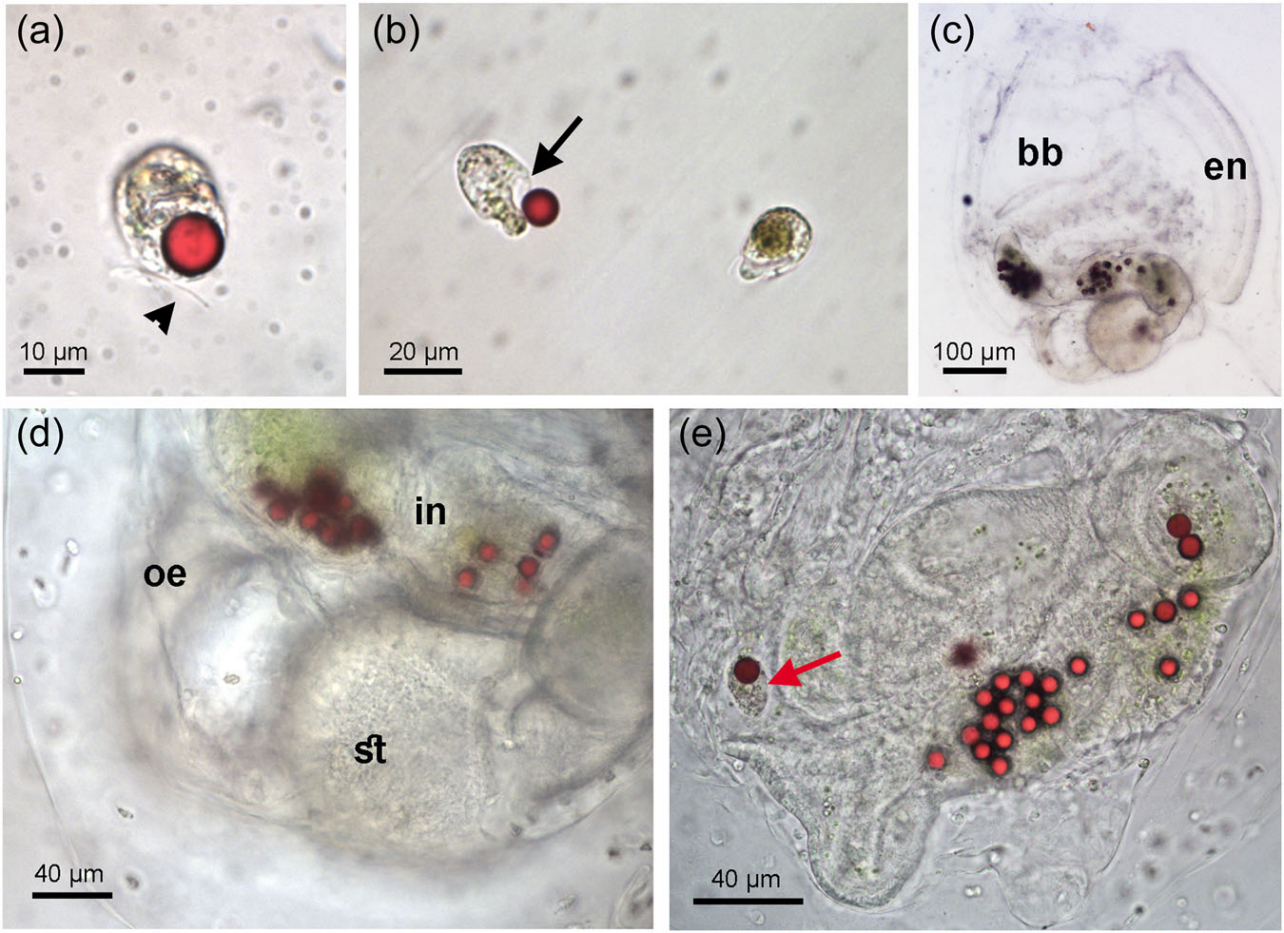
(a and b) Flagellates exposed to polystyrene microbeads. (a) A single bead is clearly visible inside the cell localized near the insertion point of the flagella. Arrowhead indicates a flagellum. (b) A cell is ingesting a microbead. The gullet is visible and indicated by a black arrow. (c)–(e) *Ciona intestinalis* juveniles exposed to microplastics (MPs). (c) Whole‐mount sample in which MPs are present in the entire digestive tract. Bb, branchial basket; en, endostyle. (d) Magnification of the digestive tract. Oe, esophagus; st, stomach; in, intestine. (e) Magnification of the digestive tract of a juvenile exposed to MPs plus flagellates. In the esophagus is present a MPs still inside the microorganism indicated by a red arrow.

Cryptomonads flagellates efficiently ingested 10 μm MPs; after 30 min of exposure 61% of cells had ingested red MPs, as shown by FACS analysis. The observed ingestion rate seems to indicate that all flagellates could be able to ingest a MP in less than 1 h. Unfortunately, this condition was not observed because exposure observation lasted 30 min. Under a transmission microscope, it was possible to observe MPs inside the cells. We never observed more than one bead inside each cell. The MPs were always localized near the point of insertion of the flagella (Figure 1a). In some cases, we were able to observe the cell ingesting MPs at the “gullet” (Figure 1b). The fate of the ingested MP within flagellate cells was not further analyzed thus we do not know how long they were retained into the protists.

### 3.2 Trophic transfer

*C. intestinalis* juveniles exposed to MPs with or without flagellates in the exposure medium showed MPs inside their digestive tract after 1.5 h of exposure (Figure 1c,d). Thanks to the transparency of ascidian tissues, it was possible to accurately count the number of ingested particles. Moreover, in juveniles exposed to MPs in the medium with flagellates, it was possible to observe the MPs still inside the protists in the first tract of the digestive system, the esophagus, whereas in the terminal part of the digestive system the beads were free in the intestine lumen (Figure 1e).

**Figure 2:**
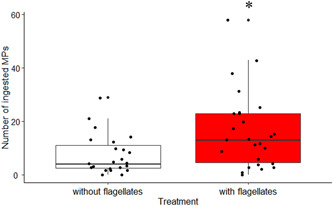
Number of microplastics (MPs) counted in *Ciona intestinalis* juveniles after exposure in seawater with or without flagellates. The number of MPs ingested by ascidians in seawater with flagellates was significantly higher compared to that of conspecifics cultured in seawater without flagellates (generalized linear mixed‐effects model followed by likelihood ratio test; *p = 0.014).

The average number of MPs in the digestive tract of juveniles exposed to microbeads alone was 7.5 versus 16.2 MPs in juveniles co‐exposed to the MPs and the flagellates (Figure 2). The 25th, 50th (median), and the 75th percentile of the number of MPs in the digestive tract of ascidian juveniles exposed to microbeads alone were 2.5, 4, and 11 MPs, respectively, while in juveniles co‐exposed to MPs and flagellates were 4, 13 and 23 MPs, respectively.

Statistical analysis by generalized linear mixed‐effects model confirmed that the difference between treatments was significant (LRT = 5.970; *p* = 0.014).

## 4 DISCUSSION

In the last few years, the impact of plastic and microplastic pollution on ecosystems has been well‐recognized. Although not many studies have explored trophic transfer of MPs (Athey et al., 2020; Batel et al., 2016; Mateos‐Cárdenas et al., 2019), this issue is gaining increasing attention (Yu et al., 2020). Particularly, MP transfer through multiple trophic levels has been reported (Setälä et al., 2014) while plastic bioaccumulation and biomagnification along the food web have never been clearly demonstrated. Thus, in this study, we use ascidian juveniles to investigate trophic transfer at the lower level of the marine food chain, to test whether the presence of flagellates ingesting MPs could affect MP concentration in ascidians.

The ability to ingest plastic microspheres by unicellular planktonic organisms, such as ciliates and flagellates, is well‐known (Athey et al., 2020; Børsheim, 1984; Stienbarger et al., 2021; Nygaard et al., 1988). It has been recently demonstrated that tintinnid ciliates, *Favella* spp, can actively ingest 10–20 µm MPs, consuming up to 10 MPs in an hour (Athey et al., 2020; Stienbarger et al., 2021). Similarly, we observed that cryptomonad flagellates actively ingest MPs. In our case, only one bead per cell was found in each flagellate, probably because of steric reasons: the volume of the MPs was almost the same as the volume of the cells. The calculated ingestion rate for the flagellates (1.2 MPs cell^−1^ h^−1^) was consistent with that reported for *Cryptomonas ovata* and *C. erosa* (0.3–2.57 MPs cell^−1^ h^−1^) exposed to 0.57 μm polystyrene microspheres in culture conditions designed to optimize phagotrophy (Tranvik et al., 1989). In our exposure conditions, about 60% of flagellates ingested MPs in 30 min, suggesting that cryptomonads can perform very efficient phagocytosis. Indeed, after 1 h of exposure, only 44% of *Favella* spp. was found to contain MPs, even if the concentration was 5 × 10^5^ particles/ml, higher than that used in this study (1000 MPs/ml). This difference could be also explained by the type of plastic or the experimental conditions that were used (Athey et al., 2020). In addition, the percentage of flagellates containing MPs could be underestimated, as it has been reported that ingested beads can be regurgitated during the fixation method used to estimate flagellate ingestion rate (Sieracki et al., 1987). However, our observations confirmed that mixotrophic cryptomonads can efficiently phagocyte 10 µm plastic particles. Moreover, by direct observation of flagellates ingesting MPs at the gullet, we can suggest that this peculiar structure, whose function has never been confirmed, is indeed involved in phagotrophic processes.

Given that there is rising concern regarding environmental contamination by MPs, our results appeared especially important because mixotrophic flagellates might be key players in the transport of MPs to higher trophic levels. This is particularly true in those environments where light is scarce and cryptomonads are abundant, since their phagotrophy rate could change according to light conditions, being higher at lower light intensity (Porter, 1988). Future works should seek to better understand the ability of unicellular organisms to ingest MPs, as already suggested in recent studies (Athey et al., 2020; Stienbarger et al., 2021).

We exposed *C. intestinalis* juveniles to MPs alone and together with cryptomonads and found that the presence of flagellates increased MPs concentration in ascidian digestive tract. Although the chosen concentration of MPs was far from environmental ones, these data were obtained in highly controlled conditions to administer an equal number of microplastics in the two treatments; the only difference was that in the first treatment the MPs were suspended in the water, whereas in the other one they were partially ingested by the flagellates. As showed by flow cytometry analysis, at the beginning of the exposure about 60% of flagellates had ingested the MPs. During exposure, most probably this percentage increased since we observed a high ingestion rate. The average number of beads in the digestive tract of ascidians doubled when the MPs had previously been phagocyted by microorganisms. This increase could be due to an augmented ingestion rate possibly due to selective capabilities of the ascidian juveniles, who might prefer to ingest algae and organic matter rather than naked plastic beads. Ascidians use the first tract of the digestive system, called branchial basket, to retain food particles suspended in the water column; the water enters from the oral syphon, goes through the stigma of the pharynx, and comes out of the organism through the atrial syphon. On the tentacles surrounding the oral syphon, mechanoreceptors called coronal organs were described and they were proposed to have a role in the selection of food (Manni et al., 2006). We can not rule out this hypothesis even if it needs to be further tested since we experimentally considered only the number of MPs in the gut and not the ingestion rate. Another possible explanation could be that the MPs had a longer residence time in the intestine of ascidians when ingested by flagellates, as the digestive process could slow down intestinal transit. Comparable results were observed in another prey‐predator pairing. Larvae of *Centropristis striata* were found to ingest a significantly higher number of MPs when fed with ciliates containing MPs than when they were directly exposed to MPs. In this case, sight could be a crucial factor for the results, as fish larvae are visual predators (Stienbarger et al., 2021).

Trophic transfer has been already documented in some marine invertebrates. Under laboratory conditions, ephyrae of *Aurelia* sp. ingested *Trigriopus fulvus* nauplii containing MPs (Costa et al., 2020). Similarly, MPs transfer could occur from copepods to mysid shrimps (Setälä et al., 2014). Indeed, zooplankton seemed to be highly affected by MPs contamination but data about MP transfer at the foundation of the marine food web are still scarce. Understanding the impact of MP transfer across all biological levels is essential for proper risk assessment and to evaluate the effects at the ecosystem level (Zara et al., 2019). In this context, our results add precious information about the routes of MPs in the environment. This study experimentally demonstrated for the first time that microplastic bioaccumulation occurs in the lower levels of the marine trophic chain, enlightening the importance of mixotrophic flagellates in MP trophic transfer. Moreover, results from this study prompt the need to trace the routes of MPs inside the environment by considering unicellular organisms that can be key actors in the process of trophic transfer at the microscale level.

## CONFLICTS OF INTEREST

The authors declare no conflicts of interest.

## Data Availability

The data that support the findings of this study are available from the corresponding author upon reasonable request.
